# Supplementary material for: Subjective and objective difficulty of emotional facial expression perception from dynamic stimuli
Source: PLoS One. 2022 Jun 16;17(6):e0269156. doi: 10.1371/journal.pone.0269156 (PMC9202844; doi:10.1371/journal.pone.0269156)
Supplement: S1 File — (DOCX) [file pone.0269156.s001.docx]

**Supplementary Materials**

| Table S1  *Emotion Categories of Facial Expression Stimuli Used in the Study* | |
| --- | --- |
| Original German term | English translation |
| amüsiert | amused |
| angeekelt | disgusted |
| angstvoll | fearful |
| ärgerlich | angry |
| beleidigt | offended |
| betroffen | affected |
| beunruhigt | troubled |
| dankbar | grateful |
| eifersüchtig | jealous |
| enthusiastisch | enthusiastic |
| entschuldigend | apologetic |
| entsetzt | aghast |
| enttäuscht | disappointed |
| erleichtert | relieved |
| erwartungsvoll | expectant |
| frustriert | frustrated |
| gekränkt | aggrieved |
| gelangweilt | bored |
| heiter | happy |
| interessiert | interested |
| melancholisch | melancholic |
| mitleidig | compassionate |
| neidisch | envious |
| neugierig | curious |
| schuldig | guilty |
| schwärmerisch | lyrical |
| stolz | proud |
| traurig | sad |
| überrascht | surprised |
| verachtend | contemptuous |
| vergebend | pardoning |
| verlegen | embarrassed |
| verliebt | in love |
| verwirrt | confused |
| verzweifelt | desperate |
| wehmütig | wistful |
| wütend | furious |
| zufrieden | content |
| zuversichtlich | confident |
| zweifelnd | doubtful |

**Supplementary equations:**

| $Rating points_{ijk}=\beta_{0}+\beta_{0}participant_{i}+\beta_{0}video_{k}+\beta_{1}*\left( actor sex_{j} \right)+\epsilon_{ijk}$ | | | | (8) | |
| --- | --- | --- | --- | --- | --- |
| $Rating standard deviation_{ijk}=\beta_{0}+\beta_{0}participant_{i}+\beta_{0}video_{k}$ $+\beta_{1}*\left( actor sex_{j} \right)+\epsilon_{ijk}$ | | | | (9) |  |
| $Rating point{s on target}_{ijk}=\beta_{0}+\beta_{0}participant_{i}+\beta_{0}video_{k}$ $+\beta_{1}*\left( participant sex_{j} \right)+\epsilon_{ijk}$ | | | (10) | |  |
| $Rating standard deviation_{ijk}=\beta_{0}+\beta_{0}participant_{i}+\beta_{0}video_{k}$ $+\beta_{1}*\left( participant sex_{i} \right)+\epsilon_{ijk}$ | (11) | | | |  |
|  |  | | | |  |
| $SRD=\beta_{0}+\beta_{1}*\left( video mean valence \right)+\beta_{2}*\left( video mean valence^{2} \right)+\epsilon$ | | (12) | | |  |

| $OD=\beta_{0}+\beta_{1}*\left( video mean valence \right)+\beta_{2}*\left( video mean valence^{2} \right)+\epsilon$ | (13) |
| --- | --- |

| $SRD=\beta_{0}+\beta_{1}*\left( video mean arousal \right)+\beta_{2}*\left( video mean arousal^{2} \right)+\epsilon$ | (14) | |  |
| --- | --- | --- | --- |
|  |  | |  |
| $OD=\beta_{0}+\beta_{1}*\left( video mean arousal \right)+\beta_{2}*\left( video mean arousal^{2} \right)+\epsilon$ | | (15) | |

| Table S2  *Mixed Effects Models to Check for a Systematic Effect of Observer Sex and Observer Age on Rating Scales with a Random Effect for Video. P values are shown in the brackets next to the estimates.* 95 % Confidence Intervals are shown in square brackets. | | | | | | | |
| --- | --- | --- | --- | --- | --- | --- | --- |
|  | *Dependent variable:* | | | | | | |
|  |  | | | | | | |
|  | angry | happy | sad | disgusted | surprised | interested | fearful |
|  | (1) | (2) | (3) | (4) | (5) | (6) | (7) |
|  | | | | | | | |
| Observer female | **-3.52**^***^ (<.001) | -1.18 (.037) | **-2.62**^**^ (.001) | **-4.49**^***^ (<.001) | **-2.91**^**^ (<.001) | **-2.18**^*^ (0.006) | **-3.31**^***^ (<.001) |
|  | [-4.99, -2.06] | [-2.28, -0.07] | [-4.11, -1.12] | [-5.96, -3.03] | [-4.44, -1.38] | [-3.71, -0.65] | [-4.80, -1.83] |
|  |  |  |  |  |  |  |  |
| Observer age | -0.01 (.718) | 0.03 (.354) | -0.05 (.222) | -0.08 (.046) | -0.10 (.02) | 0.04 (.043) | 0.06 (.18) |
|  | [-0.09, 0.07] | [-0.03, 0.09] | [-0.13, 0.03] | [-0.16, -0.002] | [-0.18, -0.02] | [-0.04, 0.13] | [-0.03, 0.14] |
|  |  |  |  |  |  |  |  |
| Intercept | 36.98 (<.001) | 31.37 (<.001) | 38.15 (<.001) | 32.69 (<.001) | 48.25 (<.001) | 51.05 (<.001) | 31.69 (<.001) |
|  | [33.69, 40.26] | [28.16, 34.58] | [34.78, 41.52] | [29.54, 35.84] | [44.95, 51.55] | [47.94, 54.16] | [28.47, 34.91] |
|  |  |  |  |  |  |  |  |
| Random effects | SD | SD | SD | SD | SD | SD | SD |
| Video | 21.37 | 27.99 | 22.12 | 18.65 | 19.58 | 15.18 | 19.51 |
| Residual | 23.66 | 17.75 | 24.19 | 23.69 | 24.83 | 24.90 | 24.01 |
| Observations | 5,292 | 5,292 | 5,292 | 5,292 | 5,292 | 5,292 | 5,292 |
| Log Likelihood | -24,798.57 | -23,529.13 | -24,922.48 | -24,747.72 | -24,996.59 | -24,909.91 | -24,831.99 |
| Akaike Inf. Crit. | 49,607.14 | 47,068.25 | 49,854.97 | 49,505.44 | 50,003.17 | 49,829.83 | 49,673.98 |
| Bayesian Inf. Crit. | 49,640.01 | 47,101.12 | 49,887.84 | 49,538.31 | 50,036.04 | 49,862.70 | 49,706.85 |
| *Note:* | ^*^p<0.05; ^**^p<0.01; ^***^p<0.001 | | | | | | |

| Table S3 *Mixed Effects Models to Investigate the Influence of the Actor Sex on the Total Rating Points Assigned per Video and the Standard Deviation Across Rating Scales with Random Effects for Video and Observer.* 95 % Confidence Intervals are shown in square brackets. | | |
| --- | --- | --- |
|  | *Dependent variable:* | |
|  |  | |
|  | Total rating points | Standard deviation across rating scales |
| Fixed effects | (1) | (2) |
|  | | |
| Actor sex female | 23.93 | 2.23 |
|  | [15.24, 32.61] | [1.19, 3.26] |
|  |  |  |
| Intercept | 240.63 | 28.58 |
|  | [232.29, 248.98] | [27.74, 29.41] |
| Random effects | SD | SD |
| Video | 44.14 | 5.24 |
| Observer | 60.67 | 4.28 |
| Residual | 63.15 | 7.75 |
|  |  |  |
|  | | |
| Observations | 5,292 | 5,292 |
| Log Likelihood | -30,415.14 | -19,094.75 |
| Akaike Inf. Crit. | 60,840.27 | 38,199.51 |
| Bayesian Inf. Crit. | 60,873.14 | 38,232.38 |
|  | | |
|  |  | |
| Note: | p-values for these exploratory analyses are intentionally not provided | |

| Table S4 *Mixed Effects Models to Investigate the Influence of the Observer Sex on the Total Rating Points Assigned per Video, the Standard Deviation Across Rating Scales and the Rating Points Spent on the Target Emotion with Random Effects for Video and Observer.* 95 % Confidence Intervals are shown in square brackets. | | | |
| --- | --- | --- | --- |
|  | *Dependent variable:* | | |
|  |  | | |
|  | Total rating points | Standard deviation across rating scales | Rating points spent on target emotion |
| Fixed effects | (1) | (2) | (3) |
|  | | | |
| Participant female | -19.01 | 1.02 | 2.57 |
|  | [-31.95, -6.07] | [0.02, 2.02] | [-0.84, 5.99] |
|  |  |  |  |
| Intercept | 266.08 | 28.97 | 79.51 |
|  | [254.46, 277.70] | [28.01, 29.94] | [76.02, 83.00] |
| Random effects | SD | SD | SD |
| Video | 45.72 | 5.35 | 9.19 |
| Observer | 60.14 | 4.26 | 6.75 |
| Residual | 63.14 | 7.75 | 20.17 |
|  |  |  |  |
|  | | | |
| Observations | 5,292 | 5,292 | 911 |
| Log Likelihood | -30,424.79 | -19,101.54 | -4,116.97 |
| AIC | 60,859.57 | 38,213.09 | 8,243.95 |
| BIC | 60,892.44 | 38,245.96 | 8,268.02 |
|  | | | |
| *Note:* | p-values for these exploratory analyses are intentionally not provided | | |

| Table S5 *Pearson Correlation of Individual Participant Ratings* | | | | | | | | | | |
| --- | --- | --- | --- | --- | --- | --- | --- | --- | --- | --- |
|  | happy | sad | surprised | disgusted | angry | fearful | interested | valence | arousal | SRD |
|  | | | | | | | | | | |
| happy | 1 | -0.50 | 0.18 | -0.38 | -0.50 | -0.36 | 0.46 | 0.87 | 0.01 | -0.12 |
| sad | -0.50 | 1 | -0.01 | 0.25 | 0.32 | 0.53 | -0.15 | -0.53 | 0.03 | 0.12 |
| surprised | 0.18 | -0.01 | 1 | 0.15 | 0.02 | 0.24 | 0.45 | 0.11 | 0.43 | 0.01 |
| disgusted | -0.38 | 0.25 | 0.15 | 1 | 0.48 | 0.36 | -0.13 | -0.45 | 0.17 | 0.11 |
| angry | -0.50 | 0.32 | 0.02 | 0.48 | 1 | 0.21 | -0.15 | -0.55 | 0.16 | 0.11 |
| fearful | -0.36 | 0.53 | 0.24 | 0.36 | 0.21 | 1 | 0.01 | -0.39 | 0.30 | 0.14 |
| interested | 0.46 | -0.15 | 0.45 | -0.13 | -0.15 | 0.01 | 1 | 0.40 | 0.26 | -0.11 |
| valence | 0.87 | -0.53 | 0.11 | -0.45 | -0.55 | -0.39 | 0.40 | 1 | -0.06 | -0.10 |
| arousal | 0.01 | 0.03 | 0.43 | 0.17 | 0.16 | 0.30 | 0.26 | -0.06 | 1 | -0.02 |
| difficulty | -0.12 | 0.12 | 0.01 | 0.11 | 0.11 | 0.14 | -0.11 | -0.10 | -0.02 | 1 |
|  | | | | | | | | | | |

| Table S6 *Pearson Correlation of Video Mean Ratings* | | | | | | | | | | |
| --- | --- | --- | --- | --- | --- | --- | --- | --- | --- | --- |
|  | happy | sad | surprised | disgusted | angry | fearful | interested | valence | arousal | difficulty |
|  | | | | | | | | | | |
| happy | 1 | -0.69 | 0.19 | -0.63 | -0.73 | -0.55 | 0.65 | 0.97 | -0.02 | -0.24 |
| sad | -0.69 | 1 | -0.18 | 0.29 | 0.35 | 0.65 | -0.43 | -0.70 | -0.005 | 0.20 |
| surprised | 0.19 | -0.18 | 1 | 0.08 | -0.15 | 0.25 | 0.58 | 0.15 | 0.66 | -0.03 |
| disgusted | -0.63 | 0.29 | 0.08 | 1 | 0.61 | 0.42 | -0.40 | -0.69 | 0.24 | 0.13 |
| angry | -0.73 | 0.35 | -0.15 | 0.61 | 1 | 0.19 | -0.46 | -0.75 | 0.16 | 0.16 |
| fearful | -0.55 | 0.65 | 0.25 | 0.42 | 0.19 | 1 | -0.14 | -0.57 | 0.42 | 0.21 |
| interested | 0.65 | -0.43 | 0.58 | -0.40 | -0.46 | -0.14 | 1 | 0.63 | 0.39 | -0.19 |
| valence | 0.97 | -0.70 | 0.15 | -0.69 | -0.75 | -0.57 | 0.63 | 1 | -0.11 | -0.19 |
| arousal | -0.02 | -0.005 | 0.66 | 0.24 | 0.16 | 0.42 | 0.39 | -0.11 | 1 | -0.09 |
| difficulty | -0.24 | 0.20 | -0.03 | 0.13 | 0.16 | 0.21 | -0.19 | -0.19 | -0.09 | 1 |
|  | | | | | | | | | | |


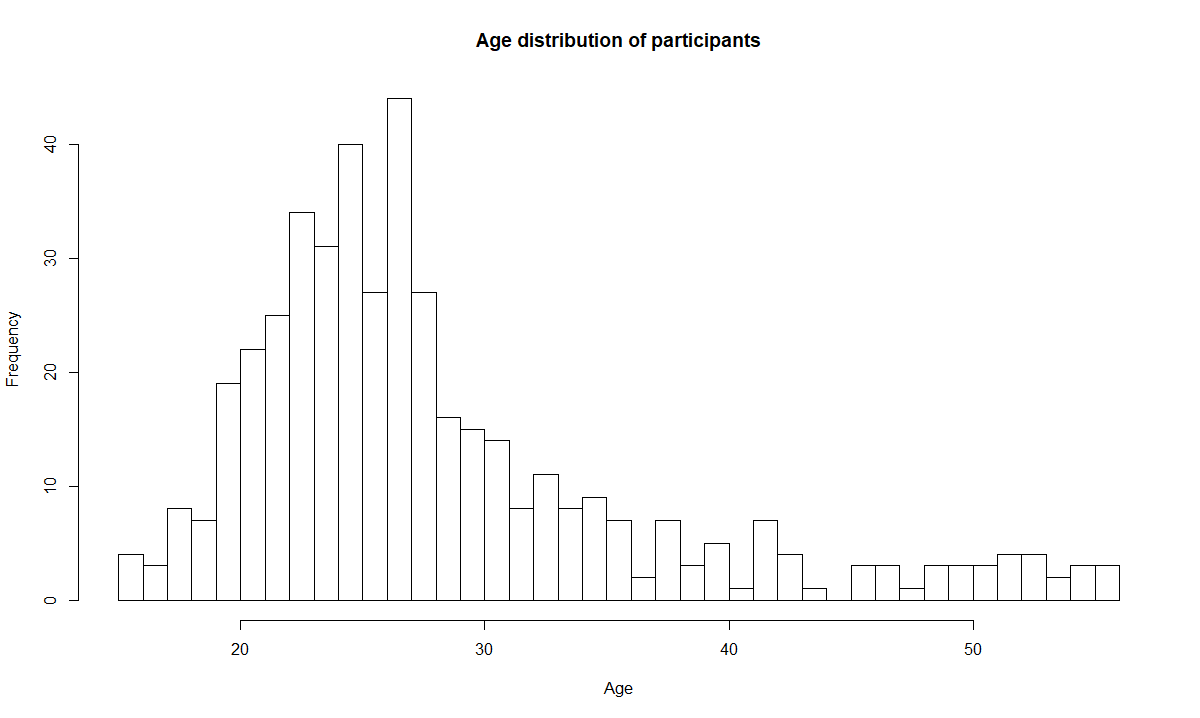


Fig S1

Histogram of age in the sample.

**Comment on the random structure of the models and study design**

To account for repeated measures, all models in our analyses include random intercepts for subjects (Hypothesis 1), videos (Hypothesis 2), or both (Hypothesis 3). In Table S7, we show this by comparing intercept-only models (M1) against model with random intercepts for subject(M2)/video(M3)/both(M4).

Following a suggestion of a reviewer, we considered whether our results can be biased by inter-subject differences. To express it in statistical terms, the models testing our hypotheses should include not only random intercepts for subjects (modelling inter-subject variance) but also random slopes for videos nested in the subjects (reflecting the interplay of subjects and stimuli). To formally test whether there is evidence for such bias in our data, below we compared null models (i.e., without fixed effects) including or not including random slopes for videos. Moreover, we additionally considered random slopes for actors.

For that, we tested whether videos (M5) or actors (M6) nested in subjects could improve the models and account for additional variance. As presented in Table S7, the addition of random slopes for videos or actors nested in subjects to null models did not significantly improve the models testing Hypotheses 1 and 3. On the other hand, models for Hypothesis 2 showed improvement with video/actor nested within subjects. However, none of the M5 or M6 models with full fixed effects structure (Max. model V or A) converged in our data. A likely reason for this is that the fixed effects already account for that variance, thus rendering our models suitable for testing the stated hypotheses.

Table S7
*Random effects structures in the models*

|  | **Subjective difficulty** | | **Objective difficulty** | |
| --- | --- | --- | --- | --- |
|  |  | *L.Ratio (p value)* |  | *L.Ratio (p value)* |
| **Hypothesis 1** | *M1 vs. M2:* | 1043.51 (<.001) | *M1 vs. M2:* | 77.05 (<.001) |
|  | *M2 vs. M5:* | <0.001 (1) | *M2 vs. M5:* | <0.001 (1) |
|  | *M2 vs. M6:* | 0.86 (.35) | *M2 vs. M6:* | 3.39 (.066) |
|  | *Max. model V:* | Not converging | *Max. model V:* | Not converging |
|  | *Max. model A:* | Not converging | *Max. model A:* | Not converging |
| **Hypothesis 2** | *M1 vs. M3:* | 114.78 (<.001) | *M1 vs. M3:* | 272.03 (<.001) |
|  | *M3 vs. M5:* | 928.73 (<.001) | *M3 vs. M5:* | 194.97 (<.001) |
|  | *M3 vs. M6:* | 929.59 (<.001) | *M3 vs. M6:* | 191.58 (<.001) |
|  | *Max. model V:* | Not converging | *Max. model V:* | Not converging |
|  | *Max. model A:* | Not converging | *Max. model A:* | Not converging |
| **Hypothesis 3** | *M1 vs. M4:* | 1043.51 (<.001) | *M1 vs. M4:* | 282.03 (<.001) |
|  | *M4 vs. M5:* | <0.001 (1) | *M4 vs. M5:* | <0.001 (1) |
|  | *M4 vs. M6:* | <0.001 (1) | *M4 vs. M6:* | <0.001 (1) |
|  | *Max. model V:* | Not converging | *Max. model V:* | Not converging |
|  | *Max. model A:* | Not converging | *Max. model A:* | Not converging |

Legend:

M1: intercept only

M2: intercept and random intercept for subject

M3: intercept and random intercept for video

M4: random intercepts for subjects and videos

M5: intercept and random slope for video nested in subject

M6: intercept and random slope for actor nested in subject

Max. model V/A: model with M5(V)/M6(A) random structure and full fixed effect structure

*Note*: All models are fit by maximizing the restricted log-likelihood. Models do not converge when there are not enough degrees of freedom to estimate correlations between slopes and intercepts.

Thus, there is little evidence for stimulus-related inter-subject bias in our data. However, one could consider eliminating this problem entirely. One way to do is to limit the total size of the stimulus set and present it to many participants. However, this would significantly constrain the range of the represented features of interest in the study: actor’s sex and age, and stimuli’s valence and arousal effects. Crucially, this would leave insufficient variance in the stimuli to reliably measure whether it is similarly difficult to perceive emotions in women and men, and we would instead measure whether it is similarly difficult for specific female and male actors. This could not be generalised to formulate population-wide interpretations, as was possible in our study.

Another possibility would be to administer a well-designed, large stimulus set including sufficient variance for the effects of interest (like the 480 videos in our study) to a (smaller) group of participants. This, however, would significantly increase duration of the experiment and fatigue experienced by the participants during the task, making ratings of emotion perception difficulty unreliable. Finally, this would also limit the range of age and sex of the observers, which were an important focus in our study. Hence, it would not be possible to reliably test the effects of (random instead of specific) observer’s gender and sex on emotion perception difficulty.

Altogether, inter-subject biases should indeed be reliably controlled for or eliminated in study designs. There are two possible ways of approaching this. One way, chosen by us in this study, is to maximise external validity by increasing the number of stimuli and characteristics of interest in order to estimate their general (and not item-specific) effects on emotion perception difficulty. The alternative approach of using a limited stimuli set would increase internal validity but would make it impossible to reliably extrapolate the results. Hence, the methodological decision here was to trade external validity for internal validity.
