# Supplementary material for: Subjective and objective difficulty of emotional facial expression perception from dynamic stimuli
Source: PLoS One. 2022 Jun 16;17(6):e0269156. doi: 10.1371/journal.pone.0269156 (PMC9202844; doi:10.1371/journal.pone.0269156)
Supplement: Attachment — Submitted filename: Response to Reviewers.pdf [file pone.0269156.s003.pdf]

Berlin, 26/03/2022

Dr. Marina A. Pavlova
Academic Editor, *PLOS ONE*

Dear Dr. Pavlova,

We thank you for giving us the chance to respond to the points raised by Reviewers about our manuscript **Subjective and Objective Difficulty of Emotional Facial Expression Perception from Dynamic Stimuli** by Jan N. Schneider, Magdalena Matyjek, Anne Weigand, Isabel Dziobek, and Timothy R. Brick. We also thank the Reviewers for taking the time to evaluate this work.

After the first round of revisions, Reviewer 1 has recommended publication of our article, but Reviewer 2 was not satisfied with our argumentation regarding a possible inter-subject bias in the light of multiple videos and actors used in our study. Specifically, the Reviewer expresses concern that since each participant rated a sub-set of a larger pool of videos (with various actors and emotional expressions), our results may be *influenced by a bias related to inter-subjects differences and therefore not valid* (direct quote). Further, the Reviewer suggests that random effects included in our models do not address this issue and that the only way to mitigate this potential bias is by administering the same small sub-set of videos to all participants. We appreciate this valid concern, and we realise that we might have not been clear enough as to why we decided for this design. We carefully reassessed our design and statistical analyses and demonstrate here additional analyses revealing that the described potential bias in our data is in fact limited and accounted for, and we explain in detail why our design is suited for maximising external validity (in contrast to trading it for internal validity, as would be the case following the Reviewer's suggestion). In the revised manuscript, we included a short explanation in the Methods section on p.15 and a comment in the Discussion of limitations on p. 31, and we further elaborated on it in the Supplementary Material (tracked changes on the last page).

Video-related or actor-related inter-subject bias in the data

The Reviewer points out that participants may present different responses to (or: be influenced differently by) various videos in our study. To express it in statistical terms, the models testing our hypotheses should include not only random intercepts for subjects (modelling inter-subject variance) but also random slopes for videos nested in the subjects (reflecting the interplay of subjects and stimuli). We agree that our models do not include the latter and thus, they make a suggestion that the stimuli influence all the participants similarly. To formally test whether there is evidence for such bias in our data, we compared null models (i.e., without fixed effects) including or not including random slopes for videos. Moreover, we additionally considered random slopes for actors. All comparisons are shown in Table 1 below (and in the revised Supplementary Material).

For example, our model for subjective difficulty in Hypothesis 1 includes random intercepts for subjects, which significantly improve an intercept-only model (M1 vs. M2). However, there is no further improvement upon addition of random slopes for videos or actors nested within subjects (M2 vs. M5/M6). This suggests that videos and actors influenced different subjects similarly. We observed analogous tendencies in all the models testing Hypotheses 1 and 3. On the other hand, models testing Hypothesis 2 showed improvement with nested videos/actors, suggesting that videos/actors influenced subjects differently. However, full models including such random structures and fixed effects cannot be estimated (convergence issues for maximum models in all comparisons). A likely reason for this is that, as we assume and explain in the manuscript for each hypothesis, the fixed effects already account for that variance, thus rendering our models suitable for testing the stated hypotheses. Nevertheless, we further considered whether the alternative approach suggested by the Reviewer would indeed be more suitable to test the hypotheses and research questions posed in our study (which are, as the Reviewer acknowledges, relevant and innovative). We discuss it in the next section.

Table 1 Random effects in the models

		Subjective difficulty		Objective difficulty
		L.Ratio (p value)		*L.Ratio (p value)*
**Hypothesis 1**	*M1 vs. M2:*	1043.51 (<.001)	*M1 vs. M2:*	77.05 (<.001)
	M2 vs. M5:	<0.001 (1)	*M2 vs. M5:*	<0.001 (1)
	M2 vs. M6:	0.86 (.35)	*M2 vs. M6:*	3.39 (.066)
	Max. model V:	Not converging	*Max. model V:*	Not converging
	Max. model A:	Not converging	*Max. model A:*	Not converging
**Hypothesis 2**	*M1 vs. M3:*	114.78 (<.001)	*M1 vs. M3:*	272.03 (<.001)
	M3 vs. M5:	928.73 (<.001)	*M3 vs. M5:*	194.97 (<.001)
	M3 vs. M6:	929.59 (<.001)	*M3 vs. M6:*	191.58 (<.001)
	Max. model V:	Not converging	*Max. model V:*	Not converging
	Max. model A:	Not converging	*Max. model A:*	Not converging
**Hypothesis 3**	*M1 vs. M4:*	1043.51 (<.001)	*M1 vs. M4:*	282.03 (<.001)
	M4 vs. M5:	<0.001 (1)	*M4 vs. M5:*	<0.001 (1)
	M4 vs. M6:	<0.001 (1)	*M4 vs. M6:*	<0.001 (1)
	Max. model V:	Not converging	*Max. model V:*	Not converging
	Max. model A:	Not converging	*Max. model A:*	Not converging

Legend:
M1: intercept only
M2: intercept and random intercept for subject
M3: intercept and random intercept for video
M4: random intercepts for subjects and videos
M5: intercept and random slope for video nested in subject
M6: intercept and random slope for actor nested in subject
Max. model V/A: model with M5(V)/M6(A) random structure and full fixed effect structure
Note: All models are fit by maximizing the restricted log-likelihood. Models do not converge when there are not enough degrees of freedom to estimate correlations between slopes and intercepts.

External vs. internal validity

To sum up the previous section, there is little evidence for the suggested stimulus-related inter-subject bias in our data. However, as the Reviewer suggests, one could aim to eliminate it entirely in the study design by administering a narrow stimulus set to all participants. This, however, would entail new problems, which we consider more severe for testing our hypotheses than the limited potential for bias discussed above.

As noted in the last revisions, there are two ways of administering the Reviewer's suggestion of the same stimulus set for each participant, which aims to maximise the internal validity of the study design and results. One is to limit the total size of the stimulus set and present it to many participants. However, this would significantly constrain the range of the represented features of interest in the study: actor's age and sex, and stimuli's valence and arousal effects. Crucially, this would leave us with not enough variance in the stimuli to reliably measure whether it is similarly difficult to perceive emotions in women and men, and we would instead measure whether it is similarly difficult for *specific* female and male actors. This could not be generalised to formulate population-wide interpretations, as was possible in our study. Another possibility would be to administer a well-designed, large stimulus set including sufficient variance for the effects of interest (like the 480 videos in our study) to a (smaller) group of participants. This, however, would significantly increase duration of the experiment and fatigue experienced by the participants during the task, making ratings of emotion perception difficulty unreliable. Finally, this would also limit the range of age and sex of the observers, which were an important focus in our study. Hence, it would not be possible to reliably test the effects of (random instead of specific) observer's gender and sex on emotion perception difficulty.

Altogether, we agree with the Reviewer's concern for inter-subject biases and we second the opinion that they should be reliably controlled for or eliminated in study designs. There are two possible ways of approaching this. One way, chosen by us in this study, is to maximise external validity by increasing the number of stimuli and characteristics of interest in order to estimate their general (and not item-specific) effects on emotion perception difficulty (see also the item-response theory; e.g., Bükner, 2019). The alternative approach of using a limited stimuli set would increase internal validity but would make it

impossible to reliably extrapolate the results. Hence, the methodological decision was to trade external validity for internal validity.

In sum, we believe that our approach is suitable for addressing the research questions stated in our study, and we sincerely hope that this detailed rebuttal offers sufficient arguments for it. We would like to thank the Reviewer once again for the thorough consideration of our work and specifically for raising this concern. Although we stand by our initial design and analysis, we highly appreciate how this comment made us reconsider and reassess our work in detail and voice the reasons for the chosen design more clearly (as stated on pages 15 and 31 in the revised manuscript, and in the Supplementary Material). We also thank you once again for considering our manuscript and we hope it is suitable for publication with *PLOS ONE*.

Yours sincerely,
Magdalena Matyjek

Email: magdalena.matyjek@hu-berlin.de
+34622282856
Clinical Psychology of Social Interaction
Berlin School of Mind and Brain
Humboldt-Universität zu Berlin
Luisenstr. 56, 10117 Berlin

Email addresses of other authors:

Jan N. Schneider
jan.schneider@hu-berlin.de

Anne Weigand
anne.weigand@hu-berlin.de

Isabel Dziobek
isabel.dziobek@hu-berlin.de

Timothy R. Brick
tbrick@psu.edu
