# Supplementary material for: Subjective and objective difficulty of emotional facial expression perception from dynamic stimuli
Source: PLoS One. 2022 Jun 16;17(6):e0269156. doi: 10.1371/journal.pone.0269156 (PMC9202844; doi:10.1371/journal.pone.0269156)
Supplement: Attachment — Submitted filename: Response to Reviewers.pdf [file pone.0269156.s004.pdf]

Berlin, 5/05/2022

Dr. Marina A. Pavlova
Academic Editor, *PLOS ONE*

Dear Dr. Pavlova,

Thank you for inviting a third Reviewer, and for allowing us to revise our manuscript, **Subjective and Objective Difficulty of Emotional Facial Expression Perception from Dynamic Stimuli**. We also thank the Reviewers for taking the time to evaluate this work. Below we reply to the latest reviews.

Comments by the Reviewer #2:
I appreciate the effort of the authors in explaining the rationale behind the experimental design they adopted. However, my concerns still remain. Especially, I understand the purpose to maximize the external validity. Nevertheless, this should not be at the expense of the sufficient level of internal validity of a study.

We are grateful to the Reviewer for their thorough consideration of our work, insightful comments, and a stimulating discussion. We regret that our arguments did not meet with fuller agreement from the Reviewer's side. As we admit, the raised concerns are valid, and we have indeed carefully considered their implications in our design and results. Nevertheless, for the reasons explained in the previous revisions and summarised in the Discussion (p. 31-32) and Supplementary Material, we stand by our work, and we hope that, if published, it will be of importance for the field.

Comments by the Reviewer #3:
Please note that I did not review the previous versions of the manuscript. Regarding the responses of authors, It is difficult to completely exclude the problem of inter-subject bias but for me the authors have done everything possible to assess this bias and to respond to the reviewer. Maybe it would be interesting to add some elements about this possible bias in the limitations but I think that it is not an argument to reject the paper. Regarding, the last version of the manuscript (R2), I find it very clear and interesting. I am not comfortable with the statistical analyzes used but the method seems in line with the objectives and the conclusions bring new important elements concerning the recognition of emotion in humans. I suggest to the authors to specify what is represented in square brackets in the tables.

We thank the Reviewer for evaluating our manuscript and for the positive feedback. As suggested, in the revised manuscript we have included a more detailed comment on the inter-subject bias limitation in the discussion (see p. 31-32). Additionally, we elaborated on it in the Supplementary Material, thus providing the additional analysis regarding the maximum structure of random effects in the models (as explained in the last replies to the Reviewers) and an extensive comment on the possible alternatives to our design and their limitations. Further, as suggested by the Reviewer, in the captions of the tables we have specified what is represented in square brackets, i.e., the 95% confidence intervals.

We thank the Reviewers for their evaluations and the Editor once again for considering our manuscript. We hope that it is now suitable for publication with *PLOS ONE*.

Yours sincerely,
Magdalena Matyjek

magdalena.matyjek@hu-berlin.de
+34622282856
Clinical Psychology of Social Interaction
Humboldt-Universität zu Berlin
Luisenstr. 56, 10117 Berlin

Email addresses of the other authors:

Jan N. Schneider: jan.schneider@hu-berlin.de

Anne Weigand: anne.weigand@hu-berlin.de

Isabel Dziobek: isabel.dziobek@hu-berlin.de

Timothy R. Brick: tbrick@psu.edu
