# Supplementary material for: Subjective and objective difficulty of emotional facial expression perception from dynamic stimuli
Source: PLoS One. 2022 Jun 16;17(6):e0269156. doi: 10.1371/journal.pone.0269156 (PMC9202844; doi:10.1371/journal.pone.0269156)
Supplement: Attachment — Submitted filename: Response to Reviewers.docx [file pone.0269156.s002.docx]

**Subjective and Objective Difficulty of Emotional Facial Expression Perception from Dynamic Stimuli**

Responses to reviews

Response to comments by Reviewer 1

**The authors investigated predictors of subjective (self-rated, SRD) and objective difficulty (OD) of emotional facial expression perception. Based on a multi-dimensional framework, they aimed to clarify the relationship between subjective / objective difficulty of emotion recognition and (i) actor’s age and sex, (ii) observer’s age and sex, and (iii) perceived valence and arousal of the stimuli. Therefore, 658 participants (German native speakers) took part in an online survey. The final dataset included 441 participants (f : m = 312 : 129; mean age: 28.08±8.17 (females) / 29.82±8.88 (males)). Each participant rated valence and arousal of 12 four-second long videos depicting dynamic facial stimuli (total set: 480 videos; 6 female / 6 male actors; mean age of actors: 35.92; 40 different emotion categories including Ekman’s six basic emotions and 34 complex emotions). Furthermore, participants provided ratings on the Basic Emotions and Interest (BEI) scales (happiness, sadness, fear, anger, surprise, disgust, and interest) and indicated subjective difficulty of making their decisions. All ratings were given on continuous visual sliding scales. Objective difficulty was defined as the Euclidean distance from an observer’s rating to the consensus of the population on a video. This consensus was calculated as the centroid in the seven-dimensional BEI rating space. Prior to hypotheses testing, a consensus check was performed following a model for each BEI item with the rating as a dependent variable and observer age and sex as independent variables. This follows the idea of a “Ground Truth” which is not predetermined by stimulus labels, but seen as the group’s consensus. The hypotheses were examined with mixed effect models and corrected for multiple testing with the Bonferroni-Holm method. In a nutshell, this work revealed (i) more dispersed ratings and higher SRD for emotions displayed by older actors, (ii) higher OD linked to female actors, (iii) a negative quadratic effect of observer age on SRD, but no quadratic effect on OD, (iv) a higher SRD and OD in female observers, but greater target emotion attribution for women, (v) a negative quadratic relationship between valence / arousal and SRD, (vi) a negative quadratic effect of valence, but a positive quadratic effect of arousal in the OD model, and (vii) a higher predictive importance of valence for both difficulty measures compared to arousal and person-specific predictors.

The strengths of the study include:

• Novel emotion perception paradigm with dynamic facial stimuli and continuous rating dimensions rather than discrete choices
• Investigation of stimuli-specific and person-specific variables of subjective and objective difficulty of emotion perception
• Insightful discussion of the “Ground Truth” concept

The presented data appear properly organized and the statistical analysis is consistent and of high methodological quality. This work includes a careful consideration of challenges and limitations of previous literature on emotion recognition, clear-cut hypotheses and a nuanced discussion of the findings. Therefore, the manuscript deserves to be published.**

Response:

We thank the Reviewer for a positive evaluation of our manuscript and pointing out the main contributions of this work.

Response to comments by Reviewer 2

**The manuscript reports findings of a study investigating predictors of subjective and objective difficulty in emotion perception from dynamic facial expressions. By administrating a novel paradigm of emotions rating, authors found that both observers-related (age, sex) and stimuli-related (age, sex, arousal, valence) variables have a predictive role on subjective/objective difficulty.
The purpose of the manuscript is relevant and innovative.
However, some important methodological issues prevent the study from the necessary rigor to be acceptable for publication. I list point-by-point my concerns.**

Response:

We thank the Reviewer for recognising the importance of our study’s focus. In the point-by-point reply we address the concerns.

**- At lines 282-283, the authors mentioned that “The minimum sample size was set to 400 participants, which would ensure that on average every one of the 480 video clips used in the study (see Materials) was rated by 10 participants …”, that means that each participant was administered a specific sub-set of stimuli, different from other subjects (about 11 videos randomly selected). This point is especially critical considering that the design of the paradigm should be cleaned by potential biases (e.g. different videos could differently influence the answer of the participant). I suggest selecting a sub-set of videos administered to all the participants (or a sub-set of participants).**

Response:

We appreciate this comment. Our decision to include a large number of video clips (480) was dictated by the fact that we were interested in generalisable effects of person- and stimulus-specific effects on difficulty in emotion perception. For this reason, we included several male and several female actors (6 each), each of which was recorded while expressing emotions spanning over 40 different categories (summing to 480 in total). This allowed us to represent different ages and genders of the actors and a wide range of expressions across the dimensions of arousal and valence. In the current design, each participant rated 12 videos (randomly chosen from the set of 480). If 12 was the total size of the stimulus set, we would not have enough variance in the stimuli to reliably measure actor’s age and sex, and stimuli’s valence and arousal effects. On the other hand, if we had 10 participants rating all the 480 videos, this would (1) significantly increase duration of the experiment and fatigue during the task, and (2) limit the range of age and sex of the observers, which were also a focus in the study. Hence, we decided for a design that maximised the ranges of the person- and stimulus-specific predictors in the study. In accordance with the Reviewer’s concern, we did anticipate that various stimuli and observers could be linked to different responses than others. For this reason, all of our regression models include random intercepts for the observers and/or videos (see section “2.5.1. Model structure” for all the models). These allowed us to statistically capture these random effects of observers and videos on the response variables (subjective and objective difficulty) and hence to appropriately estimate the effects of interest (person- and stimuli-specific predictors). Thus, we believe that our design and models are appropriate to test the hypotheses stated in the study. **- At lines 308-309 authors reported: “participants provided ratings on the Basic Emotions and Interest (BEI) scales: happiness, sadness, fear, anger, surprise, disgust, interest”. Participants rated each video on 7 emotions, 6 basic emotions, and 1 complex emotion. I suggest authors justifying the reason why BEI includes 6 basic and 1 complex emotion in the method.**

Response:

We thank the Reviewer for this important comment. The reason for choosing the 6 basic emotions as the rating scales was to conform to the literature often using these categories as response labels in emotion recognition paradigms (e.g., Kessels et al., 2014). These 6 emotion labels (happiness, sadness, fear, anger, surprise, disgust) are on one hand readily understandable and likely relatable for participants, and on the other include positive (happiness), negative (fear, anger), neutral/ambiguous (surprise and disgust) emotions of typically higher (fear) and lower (sadness) arousal. For these reasons, they are good candidates for the rating dimensions. Interest was added to this set of 6 basic emotions to represent an emotion that is considered social and more complex. Together, the dimensions based on these 7 emotions are likely a reasonable approximation of the emotion space (as we discuss on p. 30). We agree with the Reviewer that the justification for this choice was missing in the Methods. In the revised manuscript, we have included the reasons for our choice of the rating scales on p. 14.

**- The sample is not balanced in terms of gender distribution. I suggest including the observer’s gender as a covariate in the analyses (for hypotheses 1 and 3).**

Response:

We appreciate this comment. While it is true that the sample is not balanced in terms of gender (129 males and 312 females), the confirmatory analyses testing hypotheses 1 and 3 were conducted with random intercepts for observers (indirectly also including their gender). Nevertheless, following the suggestion of the Reviewer, we explored models for hypotheses 1 and 3 upon inclusion of the observer’s gender as a covariate. Tables 1 and 2 below show the results of the regression models testing hypothesis 1 and 3, respectively. For a comparison, in blue we also report the estimates and p values in the models without the gender covariate. As the tables show, both re-fitted models showed very similar estimates (effect sizes) and pattern of significance as models not including the gender covariate, which suggests that, as expected, the random intercept for observer captures the potential effects of observer’s gender. Hence, we decided not to include this additional analysis in the manuscript.

Table 1 Mixed effects models for subjective and objective difficulty predicted by actor sex and age, a covariate for observer’s sex and a random effect for observer. For comparison, in blue are reported the estimates and p values in the model without gender as a covariate. All predictors are unstandardised.

|  | *Dependent variable:* | |
| --- | --- | --- |
|  |  | |
|  | Self-rated Difficulty | Objective Difficulty |
|  | (1) | (2) |
|  | | |
| Actor female | 0.37 (0.36) | 1.86^**^ (1.84^**^) |
|  | p = .561 (.57) | p = .005 (.005) |
|  |  |  |
| Actor Age | 0.06^*^ (0.06^*^) | 0.06^*^ (0.06^*^) |
|  | p = .017 (.016) | p = .028 (.023) |
|  |  |  |
| Observer (m vs. f) | 3.57^*^ | 3.46^***^ |
|  | p = .030 | p = .0002 |
|  |  |  |
| Intercept | 35.52^***^ (38.03^***^) | 47.28^***^ (49.54^***^) |
|  | p < .001 (<.001) | p < .001 (<.001) |
|  |  |  |
|  | | |
| Observations | 5,292 | 3,537 |
| Log Likelihood | -24,335.020 | -15,504.950 |
| Akaike Inf. Crit. | 48,682.040 | 31,021.900 |
| Bayesian Inf. Crit. | 48,721.480 | 31,058.930 |
|  | | |
| *Note:* | ^*^p<0.05; ^**^p<0.01; ^***^p<0.001 | |

Table 2 Mixed effects models for self-rated and objective difficulty predicted by valence and arousal ratings, a covariate for observer’s sex, and random effects for video and observer. For comparison, in blue are reported the estimates and p values in the model without gender as a covariate. All predictors are unstandardised.

|  | *Dependent variable:* | |
| --- | --- | --- |
|  |  | |
|  | Self-rated Difficulty | Objective Difficulty |
|  | (1) | (2) |
|  | | |
| Valence | 2.45^***^ (2.44^***^) | -6.05^***^ (-6.04^***^) |
|  | p < 0.001 (<.001) | p < 0.001 (<.001) |
|  |  |  |
| Valence² | -9.30^***^ (-9.29^***^) | -1.48^***^ (-1.48^***^) |
|  | p < 0.001 (<.001) | p < 0.001 (<.001) |
|  |  |  |
| Arousal | 0.38 (0.36) | 0.70^*^ (0.71^*^) |
|  | p = 0.246 (.271) | p = 0.016 (.014) |
|  |  |  |
| Arousal² | -2.300^***^ (-2.30^***^) | 1.143^***^ (1.14^***^) |
|  | p < 0.001 (<.001) | p < 0.001 (<.001) |
|  |  |  |
| Observer (m vs. f) | 4.11^**^ | -0.7 |
|  | p = 0.009 | p = 0.448 |
|  |  |  |
| Intercept | 49.09^***^ (51.98^***^) | 56.24^***^ (55.75 ^***^) |
|  | p < 0.001 (<.001) | p < 0.001 (<.001) |
|  |  |  |
|  | | |
| Observations | 5,292 | 5,292 |
| Log Likelihood | -23,837.940 | -22,867.020 |
| Akaike Inf. Crit. | 47,693.890 | 45,752.040 |
| Bayesian Inf. Crit. | 47,753.050 | 45,811.210 |
|  | | |
| *Note:* | ^*^p<0.05; ^**^p<0.01; ^***^p<0.001 | |

**- Since the observer’s age was investigated as a predictor of subjective and objective difficulty, I believe that participants should be described also in terms of % of subjects within different ranges of age (e.g. % participants 20-30 years old, % participants 30-40 y old….)**

Response:

We agree with the Reviewer that information on age distribution of the participants (observers) should be provided. Because a histogram of age is even more informative (more detailed) than percentages for binned age categories, we have added it in the supplementary material (Fig S1) and included a reference to it in the revised manuscript (p. 11).

**- Authors should report the exact p-value in tables or in text, also for the explorative analyses.**

Response:

We thank the Reviewer for this comment. We have included the exact p values for the confirmatory analyses (testing hypotheses 1-3) reported in tables in the manuscript (Table 1 and 2) and for the consensus check in Table S2 in the supplementary material. However, we intentionally do not report p values for the exploratory analyses (as noted under each table with exploratory analyses), because their meaning is changed or lost due to unknown inflation of the alpha level (Nosek & Lakens, 2014; Wagenmakers et al., 2012).

**- The introduction is very dispersive and difficult to read, I suggest shortening it and to focus on the background supporting the three main hypotheses of the study.**

Response:

We thank the Reviewer for this comment. The introduction is intended to lead the reader from the discussion of the limitations of traditional recognition-based approaches, through the turn towards emotion perception (instead of recognition), to the main focus of the study: consideration of factors contributing to subjective and objective difficulty in emotion perception. To improve the reading flow, in the revised manuscript we have explicitly laid this out in the opening paragraphs of the Introduction (p. 3). Further, following the suggestion, we revised all the sections of the Introduction and made further improvements to make both the structure and the content easier to follow. The hypotheses are based on the brief review of so-far reported effects of age and sex of observers and actors as well as valence and arousal of the stimuli on emotion perception (as indicated in the first paragraph of the point “1.3. Hypotheses” on p. 10 in the revised manuscript), which directly precedes the section introducing the hypotheses.

**- The manuscript should be reviewed by a native-English speaker.**

Response:

We thank the Reviewer for this suggestion. Accordingly, one of the authors (T.R.B.) who is a native English speaker and an expert in the field has thoroughly checked and corrected the revised manuscript.

Cited literature:

Kessels, R. P. C., Montagne, B., Hendriks, A. W., Perrett, D. I., & de Haan, E. H. F. (2014). Assessment of perception of morphed facial expressions using the Emotion Recognition Task: Normative data from healthy participants aged 8-75. *Journal of Neuropsychology*, *8*(1), 75–93. https://doi.org/10.1111/jnp.12009

Nosek, B. A., & Lakens, D. (2014). Registered Reports. *Social Psychology*, *45*(3), 137–141. https://doi.org/10.1027/1864-9335/a000192

Wagenmakers, E.-J., Wetzels, R., Borsboom, D., van der Maas, H. L. J., & Kievit, R. A. (2012). An Agenda for Purely Confirmatory Research. *Perspectives on Psychological Science*, *7*(6), 632–638. https://doi.org/10.1177/1745691612463078
